# Strengthening Collaboration and Safety in Aesthetic Plastic Surgery: The Role of the AlliedPro Program

**DOI:** 10.1093/asjof/ojaf029

**Published:** 2025-04-22

**Authors:** Sachin M Shridharani

I am writing to express my thoughts on the recent launch and continued impact of the AlliedPro Program by the American Society for Aesthetic Plastic Surgery. This program, designed to foster deeper collaboration among aesthetic professionals, including nonphysician providers, marks a pivotal development in the landscape of aesthetic medicine. As an active participant in the field and co-chair of the program, I believe AlliedPro offers several key benefits that warrant discussion and consideration by the broader medical community.

First and foremost, the AlliedPro Program provides a platform for nonphysician professionals such as physician assistants, nurse practitioners, and registered nurses to receive specialized education and training tailored to the demands of aesthetic plastic surgery. In an era where demand for noninvasive and minimally invasive aesthetic treatments is increasing and has eclipsed the number of aesthetic surgical procedures on a per annum basis, the AlliedPro initiative helps ensure that those delivering care are equipped with up-to-date knowledge and skills. The structured educational opportunities within the program not only enhance the competency of providers but also promote patient safety and satisfaction—both critical pillars of modern aesthetic practice.

Moreover, the program facilitates a more cohesive, team-based approach to patient care. Collaboration between physicians and nonphysician providers has been shown to improve outcomes in various healthcare settings. In aesthetic surgery, where a nuanced understanding of patient expectations and surgical artistry is crucial, fostering strong interdisciplinary communication can enhance both procedural success and patient satisfaction. By establishing common ground and shared goals between surgeons and allied professionals, the AlliedPro Program strengthens the overall quality of care delivered in aesthetic practices.

Importantly, the AlliedPro Program also addresses an increasing demand for regulation and standardization in aesthetic practice. As the field continues to expand, ensuring that providers have met high standards of training and education is essential for mitigating risks associated with poorly performed treatments. By offering accredited, evidence-based training modules, the program ensures that allied professionals are not only highly skilled but also well-versed in the ethical and safety standards expected in aesthetic surgery. It is important to emphasize that this initiative is not about enabling nonplastic surgeons to perform procedures beyond their scope but rather about enhancing patient safety and care through structured, standardized education. These established educational protocols have been developed by expert clinicians over several years, rooted in evidence-based medicine, experience-based practice, and clinical trial protocols.

However, it is essential that the program be continually evaluated and adapted to address the dynamic nature of the aesthetic industry. As technology, techniques, and patient preferences evolve, ongoing research and feedback from all stakeholders—physicians, allied professionals, and patients—should guide future updates to the program's curricula. This iterative approach will ensure the AlliedPro Program remains a vital resource for maintaining excellence in the field.

In conclusion, the AlliedPro Program represents a commendable effort by The Aesthetic Society to enhance professionalism, collaboration, and education within the rapidly expanding aesthetic plastic surgery community. By fostering a well-rounded and highly skilled workforce, the program is poised to contribute significantly to the overall advancement of patient care and the sustainability of the aesthetic medicine field.

